# Player archetypes within basketball: optimizing roster composition to create a championship team

**DOI:** 10.3389/fspor.2025.1639431

**Published:** 2025-08-29

**Authors:** Luke S. J. Penner

**Affiliations:** Fowler College of Business, San Diego State University, San Diego, CA, United States

**Keywords:** NBA, FIBA, player archetypes, roster composition, talent identification, sports analytics

## Abstract

The present study assessed the feasibility of grouping university and professional basketball players across different leagues based on their playing styles to optimize championship team construction, moving beyond traditional positional constraints towards a positionless approach. A comprehensive dataset of 22,500 elite professional and university athletes from 110 leagues, sourced from EuroBasket, RealGM, and USportsHoops, was analyzed. Player performance was quantified using 13 standardized box score statistics converted to per 48 min. Utilizing the k-means algorithm, players were clustered into 9 distinct player archetypes. Multiple linear regression models were then developed for each archetype, predicting “points per minute” to facilitate player ranking, further refined by a data-driven league quality weighting system. Optimal player cluster proportions were derived from analyses of 2018/19 NBA lineups and 2014−2018 international medal-winning teams to create the most effective team line-ups. The model's utility was demonstrated by selecting a hypothetical Team Canada roster for the 2019 FIBA World Cup, which showed effectiveness in identifying a robust team composition and predicting the rise of future high-potential players. Additionally, the model's effectiveness was evaluated by comparing its results to the composition of the 2000 and 2024 Canadian National Olympic teams. The findings revealed 9 unique player clusters, demonstrating the model's potential as a valuable tool for guiding coaching decisions in drafting and signing future players to fit their roster composition effectively. The model proved to be a novel method for talent identification due to its evaluation of players across leagues and its usage of readily accessible data for coaches and scouts alike. Despite limitations related to data sources and subjective weighting, this research provides a sophisticated analytical tool for players, coaches, scouts, and general managers, offering a comprehensive league strength metric and a nuanced player ranking system to enhance roster development alongside the expertise of coaches in the evolving global basketball landscape.

## 1 Introduction

The game of basketball, invented by Dr. James Naismith in 1891, has undergone a significant evolution. Initially played with nine players per team, it transformed into a five-player format used today (1). For decades, the sport relied on traditional positions, point guard, shooting guard, small forward, power forward, and center, which were typically associated with height ranges and playing styles (2). This structure provided a clear understanding of player roles. However, the modern game, particularly influenced by the European style, is rapidly shifting towards positionless basketball, challenging these rigid constraints. Many successful European teams, like Fenerbahçe, CSKA Moscow, and Real Madrid, have revolutionized the game by prioritizing versatility and skill over fixed roles, deploying starting lineups optimized for overall team success. While existing research often focuses on NBA players, there is a clear need to expand this scope. By expanding the focus of the potential groupings of players, one may be able to find trends within youth, domestic, and professional leagues to help find the next exceptional athlete that does not fit a typical mold but rather operate effectively between traditional positions.

For coaches, embracing the potential of unconventional lineups is crucial for success in this evolving landscape. This approach is particularly relevant for national teams, such as Canada, which are experiencing an influx of NBA prospects and stars. A deeper understanding of player archetypes makes selecting talent from diverse leagues, including the NBA G-League and various European leagues, less risky and more predictable for both international success and the NBA drafting process. Cheng (3) mentioned that expanding research to include National Collegiate Athletic Association (NCAA) players could give scouts and coaches valuable benchmarks for comparison as prospects enter the NBA draft. Ultimately, the goal is to win games, which can be done by enhancing team dynamics and optimizing success by prioritizing player selection, on-court placement, and team synergy over rigid adherence to stereotypical positions.

Beyond athletic ability, personality traits also play a significant role in elite athletic performance (4–6), underscoring that cohesive teams often outperform those relying solely on individual talent. The dynamic nature of athletic development means that coaches and scouts must consider athletes' ongoing progression. As players advance, their personality traits tend to become more homogeneous (7). This homogeneity demands a nuanced approach to player evaluation that extends beyond traditional box score statistics, incorporating unique skill developments, such as the “dipping” motion in a jump shot increasing accuracy (8). Such an approach is vital for accurately discerning appropriate player archetypes, leading to optimized roster construction.

As basketball players progress towards a professional career, it is essential to assess both their potential quality, the anthropological attributes, and their actual quality, the on-court results (9, 10). In player development, the term “green banana” refers to athletes who take longer to mature, highlighting the importance of a system to identify archetypes and their developmental pathways. When a player seeks to transition from NCAA Division I to the NBA, or from one European league to another, understanding different skill growth trajectories and relevant statistics is crucial for contextualizing their actual quality against varying levels of competition. To accurately assess a player's true skill and performance in a model, particularly against weaker competition, a weighting system is necessary to score league results effectively. Drawing inspiration from the league and country ranking systems used by the International Basketball Federation (11) and the Union of European Football Associations (12), this study develops a similar system to translate player results across leagues, thereby ascertaining their true quality for higher-tier leagues or international competition. Given the evolving landscape of modern basketball and the identified need for a more nuanced approach to player evaluation, this study's overarching aim is to construct a robust, data-driven model for roster composition and talent identification across the many leagues of the world.

## 2 Methods

### 2.1 Player data

Player data was sourced from three primary platforms: https://www.usportshoops.ca, https://www.eurobasket.com, and https://www.basketball.realgm.com. Data collection focused on the 2018/19 league seasons, spanning from October 2018 to approximately June 2019. EuroBasket served as the primary data source for the majority of leagues. Exceptions, necessitated by data deficiencies in the primary source, included Puerto Rico's Baloncesto Superior Nacional, Venezuela's Liga Profesional de Baloncesto, and Iran's Super League, for which data were sourced from RealGM. Similarly, data for the Canadian University system was exclusively obtained from USports Hoops. These specific leagues were integrated into the dataset due to their significance for model development. Several countries were deliberately excluded from the dataset due to a lack of current league information.

The initial dataset encompassed 27,363 players across 110 distinct professional and collegiate leagues, including prominent competitions such as the NBA, G-League, and NCAA in North America; Liga Endesa and VTB United League in Europe; Novo Basquete Brasil in South America; and the Chinese Basketball Association and the National Basketball League in Asia. To ensure data quality and consistency, players who rarely participated were removed. A standard criterion was applied: a player was excluded if they played less than 10% of the maximum minutes logged by any player within the same league. This threshold was league-specific to prevent over-exclusion in leagues with fewer games. After applying this criterion, the final dataset comprised 22,500 athletes. The obtained sample size of 22,500 athletes substantially exceeded established benchmarks for similar research (3, 13, 14), thereby ensuring robust and generalizable findings.

### 2.2 League quality assessment

To differentiate between league quality levels, each league was ranked and subsequently divided into subgroups. Four metrics were employed to assess league quality: average player height, a win quality weight, 3-point percentage, and free throws attempted per field goal made. These metrics were chosen as indicators of strong teams, particularly the latter two (15). Player height within leagues was considered indicative of player potential (M. Meeks & P. Jevtovic, personal communication, April 14, 2019).

The win quality metric was developed by assigning weights to each competition, in a manner analogous to the UEFA league ranking system. To capture high-level competition, only post-season games for NBA and G-League playoffs were considered. For university leagues, the National tournaments of “March Madness Division I and II” and the “Elite 8” were included. For leagues outside of North America, major international club tournaments were considered, including: EuroLeague, EuroCup, Basketball Champions League, and the Europe Cup in Europe; the Asia Champions League, “Terrific 12”, and “Summer 8” in Asia; and the Americas League, and South American League in South America. Multipliers for each competition were established based on discussions with Canadian national team analysts (M. Meeks & P. Jevtovic, personal communication, April 14, 2019) and informed by the UEFA model's weighting of competitions, where Champions League was valued more than Europa League.

To calculate the win quality metric, each team was assigned a score based on its participation and progression within a given tournament, including bonus points for advancing in the competition, which was then multiplied by the corresponding tournament multiplier. For example, a team making the EuroLeague quarterfinals would receive points for EuroLeague participation, a reduced number of points for wins to mitigate win inflation, and bonus points for advancing to the Round of 16. This sum was then multiplied by the designated multiplier such as 1.9 for the EuroLeague. Once all teams were scored, a “Continental Competition Average” was established by averaging all team scores within a given country. This metric primarily reflected top-tier leagues and teams that participated in these championships. Leagues were subsequently ranked based on their composite win quality metric. Based on these rankings, leagues were divided into divisions. The NBA and G-League were designated as separate divisions. For other continents, divisions were created by calculating the mean and standard deviation of the ranking metrics. Europe required two additional divisions, “Europe A”, “Europe B”, and “Europe C”, due to the distribution of scores, resulting in groups of approximately 15%, 55%, and 30% of teams. Other continents like the Americas, Asia, and University level, were divided into two divisions each, separating the top 30% of leagues from the remaining 70%. This division methodology aimed to provide meaningful separation between leagues while ensuring inclusion of players meeting the 10% playing time requirement, regardless of their team's playoff or tournament qualification.

### 2.3 Variable selection for clustering and regression analyses

Player on-court performance, encompassing both positive and negative contributions, was quantified through conventional box score statistics. The variables selected were: points per game (PTS), offensive rebounds per game (OREB), defensive rebounds per game (DREB), assists per game (AST), blocks per game (BLK), steals per game (STL), personal fouls per game (PF), free throw attempts per game (FTA), free throw percentage (FT%), 2-point field goal attempts per game (2PA), 2-point field goal percentage (2P%), 3-point field goal attempts per game (3PA), and 3-point field goal percentage (3P%). All statistics represented season averages collected at the conclusion of the respective seasons. Playoff statistics were explicitly excluded due to potential biases introduced by reduced sample sizes and observed alterations in playing style, particularly in series-based competitions.

For player clustering, the absolute values were standardized to a per 48 min basis to account for varying playing times. This standardization was applied to all standard box score variables, except for field goal attempts and percentages, which were separated into 2-point and 3-point categories to evaluate shot location. The variables “minutes per game” (MIN) and “turnovers per 48 min” (TOV) were omitted from the clustering variable set. Minutes per game was excluded to prevent confounding player performance metrics with playing time, while turnovers per 48 min was unavailable in the EuroBasket dataset. This variable selection aligns with established methodologies in prior research (16, 17).

For the subsequent player ranking within identified clusters, a modified set of variables was utilized. The primary modification involved the replacement of “points per game per 48 min” with “minutes per game”. This adjustment was imperative as “points per minute” served as the dependent variable in the regression analyses, and its inclusion as an independent variable was necessary. A critical aspect of this analysis involved the independent regression analysis of each cluster, which yielded a distinct regression model for each archetype. The systematic removal of non-significant variables within each cluster aimed to identify the most impactful predictors for each distinct player archetype. The decision to rank players based on “points per minute” was based on the fundamental objective of basketball. The goal was to maximize points scored, thereby ensuring that the selected archetypes not only contributed cohesively to team composition but also maximized scoring efficiency.

### 2.4 Data analysis

The data collection process was meticulously designed to ensure replicability by other researchers. Leagues were excluded if they lacked statistics on EuroBasket or RealGM, were youth leagues (under-21 or under-19), or were classified as amateur or semi-professional leagues. To facilitate standardized comparisons of player performance, all raw data were converted into per 48 min statistics.

Player clustering was executed utilizing the k-means algorithm. To mitigate the disproportionate influence of variables with larger magnitudes on the clustering outcomes, all variables were standardized using z-scores. The optimal number of clusters was determined using the Within-Cluster Sum of Squares (WSS) method, with the selected number of clusters corresponding to the flattest region of the WSS plot (14), or an “elbow” in the data. This decision was further supported by prior research (18). The similarity of clustering across different league subsets was assessed using the Jaccard coefficient of similarity (see Equation 1), where “a” represented the number of players clustered in the same cluster, and “b” and “c” represented players not clustered correctly.

$$Jaccard\;Coefficient=\;\frac{a}{a+b+c}$$ (1)

An analysis of variance (ANOVA) was subsequently conducted to assess the statistical significance of each variable in differentiating between the derived clusters. A Tukey's Honestly Significant Difference (HSD) test was employed to identify the specific variables contributing to significant differences between cluster pairs, using a studentized range q table at *α* = 0.05, critical q-value = 4.387.

Multiple linear regression models were then developed for each identified player cluster, with “points per minute” as the dependent variable. Prior to fitting the models, comprehensive diagnostic tests were performed to validate the underlying assumptions of linear regression. These tests included the Shapiro–Wilks test for assessing the normality of the data set, the Durbin-Watson test for detecting autocorrelation among residuals, and the calculation of the Variance Inflation Factor (VIF) to identify potential multicollinearity among the independent variables. A stricter VIF cutoff of 3 was applied for caution, with variables exceeding this threshold being removed iteratively. Outliers were identified using Cook's distance, with values above 1 being considered outliers.

To enhance the model's quality, “University A” was further subdivided into three tiers: “Major”, “Mid-Major”, and “Small”, following a categorization approach similar to Reinig and Horowitz (19). Each cluster's regression model was refined by retaining only statistically significant variables, resulting in unique regression lines for each cluster. These lines were then used to predict player points per minute, enabling a relative ranking within each cluster. To account for league quality, a weighting system was applied to these rankings, influenced by data-driven insights and discussions with Canadian national team coaches (M. Meeks, & P. Jevtovic, personal communication, March 22, 2019).

To identify the optimal player cluster proportion for national team composition, NBA lineups from the 2018/19 season and all medal-winning athletes from international competitions from 2014 to 2018 were analyzed. Players were selected if they had a plus-minus of at least 15 and played at least 10% of the maximum minutes. The selection of Canadian players was determined primarily by the overall proportion derived from both groups. However, if two clusters exhibited close proximity, the NBA proportion was prioritized.

## 3 Results

### 3.1 Demographics

The model data set comprised 22,500 elite university and professional basketball athletes across 110 leagues (see Table 1). Player positions were categorized as guard, forward, center, guard-forward, or forward-center. The professional player distribution included 6,901 guards, 4,942 forwards, 1,564 centers, 1,221 guard-forwards, and 1,154 forward-centers. For university players, the distribution was 3,740 guards, 2,263 forwards, 276 centers, 303 guard-forwards, and 136 forward-centres.

**Table 1:** Demographics of the professional and university basketball players.

Player position		Professional players			University players			
	Factor	*n*	Average	Standard deviation	*n*	Average	Standard deviation	Frequency
Guards	*n*	6,901			3,740			
	Age	–	26.0	5.0	–	–	–	–
	Height	–	187.7 cm	6.2 cm	-	188.8 cm	6.0 cm	–
		–	6′1.9″	2.4″	-	6′2.3″	2.4″	–
	Freshman	–	–	–	–	–	–	827
	Sophomore	–	–	–	–	–	–	838
	Junior	–	–	–	–	–	–	1,096
	Senior	–	–	–	–	–	–	979
Forwards	*n*	4,942			2,263			
	Age	–	26.5	4.9	–	–	–	–
	Height	–	199.3 cm	6.3 cm	–	201.1 cm	4.3 cm	–
		–	6′6.5″	2.5″	–	6′7.2″	1.7″	–
	Freshman	–	–	–	–	–	–	471
	Sophomore	–	–	–	–	–	–	546
	Junior	–	–	–	–	–	–	619
	Senior	–	–	–	–	–	–	627
Centres	*n*	1.564			276			
	Age	–	27.1	5.0	–	–	–	–
	Height	–	206.7 cm	5.1 cm	–	207.6 cm	5.2 cm	–
		–	6′9.4″	2.0″	–	6′9.7″	2.1″	–
Guard-Forwards	Freshman	–	–	–	–	–	–	52
	Sophomore	–	–	–	–	–	–	49
	Junior	–	–	–	–	–	–	82
	Senior	–	–	–	–	–	–	93
	*n*	1,221			303			
	Age	–	27.1	5.0	–	–	–	–
	Height	–	206.7 cm	3.9 cm	–	196.5 cm	3.6 cm	–
		–	6′9.4″	1.5″	–	6′5.4″	1.4″	–
	Freshman	–	–	–	–	–	–	71
	Sophomore	–	–	–	–	–	–	79
	Junior	–	–	–	–	–	–	81
	Senior	–	–	–	–	–	–	72
Forward-centres	*n*	1,154			136			
	Age	–	28.2	5.0	–	–	–	–
	Height	–	204.2 cm	3.9 cm	-	204.8 cm	4.7 cm	–
		–	6′8.4″	1.5″	-	6′8.6″	1.9″	–
	Freshman	–	–	–	–	–	–	30
	Sophomore	–	–	–	–	–	–	36
	Junior	–	–	–	–	–	–	41
	Senior	–	–	–	–	–	–	29

Note: Total *n* = 22,500.

League quality levels were differentiated using a ranking system based on average player height, a win quality metric, 3-point percentage, and free throws attempted per field goal made. In the context of leagues across the world, player height within leagues was indicative of player potential, and thus, leagues like Liga Endesa, a top European league had a higher player average height at 199.00 cm compared to the Latvian Basketball League Division 2, a lower European league, who had a player average height of 189.67 cm (see Table 2). For win quality, the NBA exhibited the highest win quality metric at 262.15, followed by Liga Endesa at 217.31. Based on these metrics, leagues were categorized into distinct divisions: NBA, G-League, “Europe A”, “Europe B”, “Europe C”, “Americas A”, “Americas B”, “Asia A”, “Asia B”, “University A”, and “University B”.

**Table 2:** League ranking multiplier.

League	Weight
NBA	1.10
G-League	0.85
Europe Pro A	0.90
Europe Pro B	0.70
Europe Pro C	0.50
America Pro A	0.65
America Pro B	0.55
University A Major	0.70
University A Mid-Major	0.65
University A Small	0.50
University B	0.60

### 3.2 Clustering the athletes

The optimal number of clusters was determined to be 9. An initial assessment of clustering similarity across individual league groupings using the Jaccard coefficient of similarity yielded a value of 11.89%, indicating low similarity. The entire data set was clustered together, resulting in 9 distinct player archetypes across the leagues.

Analysis of Variance (ANOVA) revealed that all variables utilized in the clustering process were statistically significant in differentiating between the identified athlete clusters (see Table 3). Subsequently, a Tukey's HSD *post hoc* test further identified these distinctions. Out of 936 pairwise comparisons across the 13 variables and 9 clusters, 864 pairs demonstrated statistically significant differences (see Table 4). The majority of the 72 statistically insignificant pairs were concentrated within the free throw percentage, 2-point percentage, and 3-point percentage variables, with 2-point percentage contributing 27 of these pairs.

**Table 3:** Variables significance for clustering.

Factor	Cluster		Error		F-value	*p*-value
	Mean square (between)	df	Mean Square (within)	df		
PTS/48	1573.434	8	0.441	22,491	3,569.998	0.000
ORB/48	1,646.799	8	0.415	22,491	3,971.587	0.000
DRB/48	1,265.447	8	0.550	22,491	2,299.647	0.000
AST/48	1,479.513	8	0.474	22,491	3,120.393	0.000
BLK/48	1,691.467	8	0.399	22,491	4,241.841	0.000
STL/48	948.376	8	0.663	22,491	1,430.324	0.000
PF/48	714.582	8	0.746	22,491	957.623	0.000
FTA/48	1,232.493	8	0.562	22,491	2,193.048	0.000
FT%	973.484	8	0.654	22,491	1,488.233	0.000
2PA/48	1,576.098	8	0.440	22,491	3,583.744	0.000
2P%	558.035	8	0.802	22,491	695.907	0.000
3PA/48	1,424.025	8	0.494	22,491	2,883.353	0.000
3P%	1,172.085	8	0.583	22,491	2,008.768	0.000

Note: PTS/48 = points per 48 min, ORB/48 = offensive rebounds per 48 min, DRB/48 = defensive rebounds per 48 min, AST/48 = assists per 48 min, BLK/48 = blocks per 48 min, STL/48 = steals per 48 min, PF/48 = personal fouls per 48 min, FTA/48 = free throws attempted per 48 min, FT% = free throw percentage, 2PA/48 = 2 -point attempted per 48 min, 2P% = 2-point percentage, 3PA/48 = 3-point attempted per 48 min, 3P% = 3-point percentage.

**Table 4:** Pairwise comparison significance.

Factor	Insignificant pairs													
PTS/48	4-5	–	–	–	–	–	–	–	–	–	–	–	–	–
	–	–	–	–	–	–	–	–	–	–	–	–	–	–
ORB/48	–	–	–	–	–	–	–	–	–	–	–	–	–	–
	–	–	–	–	–	–	–	–	–	–	–	–	–	–
DRB/48	1-2	1-9	2-9	–	–	–	–	–	–	–	–	–	–	–
	–	–	–	–	–	–	–	–	–	–	–	–	–	–
AST/48	1-2	1-7	2-7	6-7	–	–	–	–	–	–	–	–	–	–
	–	–	–	–	–	–	–	–	–	–	–	–	–	–
BLK/48	1-2	2-9	3-6	–	–	–	–	–	–	–	–	–	–	–
	–	–	–	–	–	–	–	–	–	–	–	–	–	–
STL/48	1-2	1-4	1-5	2-4	2-5	4-5	5-8	6-9	–	–	–	–	–	–
	–	–	–	–	–	–	–	–	–	–	–	–	–	–
PF/48	2-9	–	–	–	–	–	–	–	–	–	–	–	–	–
	–	–	–	–	–	–	–	–	–	–	–	–	–	–
FTA/48	3-9	4-9	–	–	–	–	–	–	–	–	–	–	–	–
	–	–	–	–	–	–	–	–	–	–	–	–	–	–
FT%	2-6	2-9	3-4	3-5	3-7	4-5	4-7	4-9	5-7	5-8	6-9	–	–	–
	–	–	–	–	–	–	–	–	–	–	–	–	–	–
2PA/48	–	–	–	–	–	–	–	–	–	–	–	–	–	–
	–	–	–	–	–	–	–		–	–	–	–	–	–
2P%	1-2	1-3	1-9	2-3	2-4	2-6	2-8	2-9	3-4	3-5	3-6	3-7	3-8	3-9
	4-5	4-6	4-7	4-8	4-9	5-6	5-7	5-8	6-7	6-8	6-9	7-8	8-9	–
3PA/48	–	–	–	–	–	–	–	–	–	–	–	–	–	–
	–	–	–	–	–	–	–	–	–	–	–	–	–	–
3P%	1-3	1-7	1-9	2-4	2-6	2-9	3-7	3-9	4-6	4-9	5-8	6-9	–	–
	–	–	–	–	–	–	–	–	–	–	–	–	–	–

Note: Critical q-value = 4.387.

**Figure 1:**
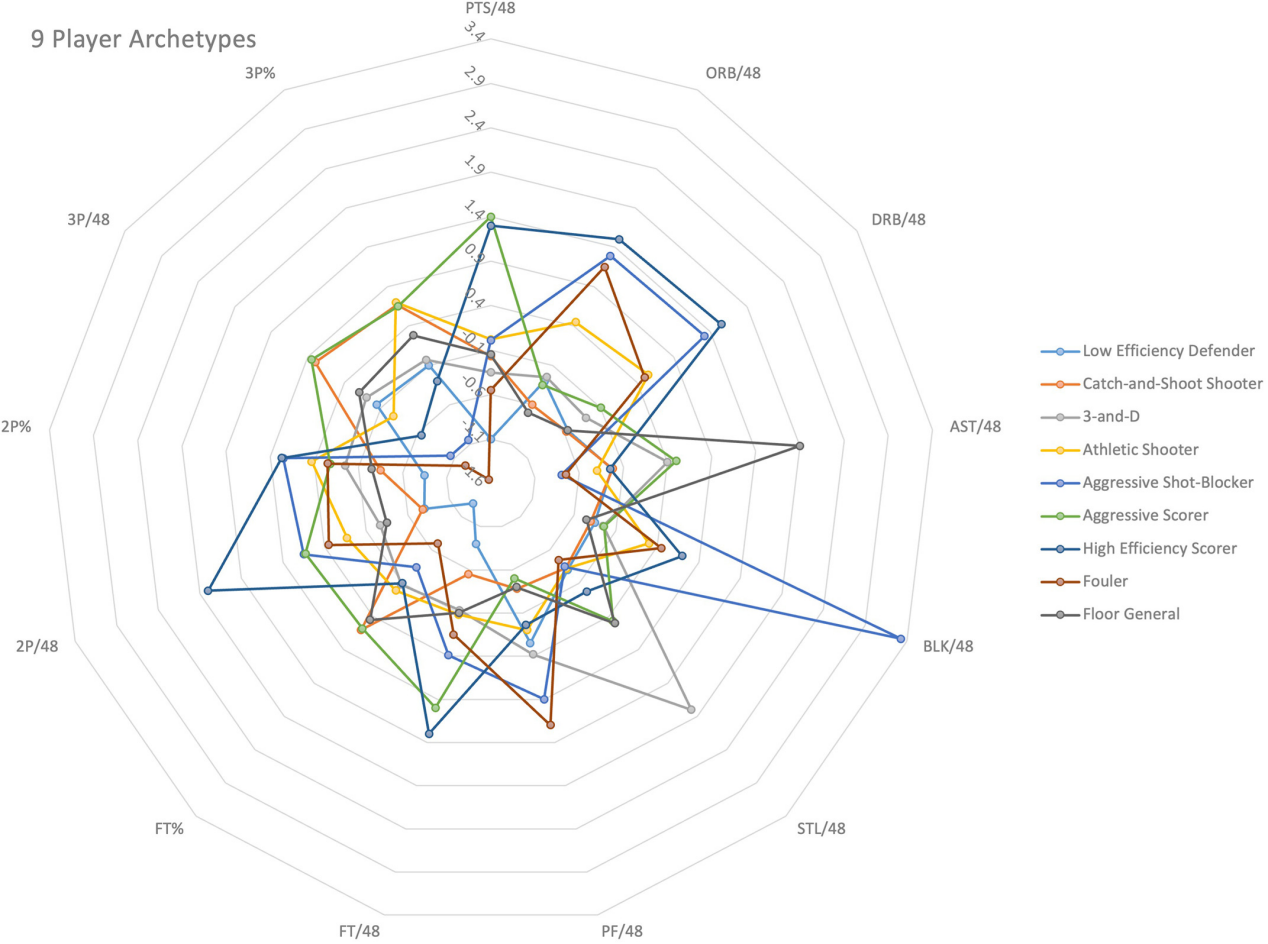
Radar graph illustrating the 9 Player Archetypes and the 13 box score statistics used to determine the categorization.

The 9 resulting clusters were assigned descriptive labels reflecting their predominant skill sets: “Low Efficiency Defender”, “Catch-and-Shoot Shooter”, “3-and-D”, “Athletic Shooter”, “Aggressive Shot-Blocker”, “Aggressive Scorer”, “High Efficiency Scorer”, “Fouler”, and “Floor General” (see Table 5). The distinct separation of these clusters was visually supported (see Figure 1).

**Table 5:** Characterization of the archetypes.

Cluster	Archetype	Characteristics			Example player
1	Low Efficiency Defender	Low 2PA/48	Low 2P%	High PF/48	OG Anunoby
2	Catch-and-Shoot Shooter	High 3PA/48	Low 2PA/48		Eric Gordon
3	3-and-D	High STL/48	High 3PA/48		Robert Covington
4	Athletic Shooter	High 3P%	High TRB/48		Nikola Mirotic
5	Aggressive Shot-Blocker	High BLK/48	High PF/48	High TRB/48	JaVale McGee
6	Aggressive Scorer	High PTS/48	High FTA/48	High 3PA/48	James Harden
7	High Efficiency Scorer	High 2PA/48	Low 3PA/48		Giannis Antetokounmpo
8	Fouler	High PF/48	Low STL/48		Jon Leuer
9	Floor General	High AST/48	High 2P%	High 3P%	Chris Paul

### 3.3 Ranking the athletes in each archetype

Player ranking within each identified archetype was performed using multiple linear regression models designed to predict “points per minute”. Prior to model fitting, diagnostic tests for normality, autocorrelation, and multicollinearity were conducted. The Shapiro–Wilks test indicated that the “points per minute” variable did not exhibit a normal distribution (see Table 6); however, given the substantial sample size, regression analysis proceeded. Variance Inflation Factors (VIFs) were calculated to assess multicollinearity. The final models exhibited low multicollinearity, characterized by a mean VIF of 1.160 across all clusters and a maximum VIF of 1.549 for “minutes per game” in Cluster 8. Durbin-Watson statistics, used to assess autocorrelation, generally fell within the acceptable range of 1.5–2.5 for most clusters, with Cluster 1 being an exception. Outlier detection via Cook's distance identified only 1 outlier, Marcin Chodkiewicz from Europe Pro C, across the entire dataset, which was retained due to the large sample size.

**Table 6:** Regression diagnostic tests.

Cluster	Shapiro–Wilks				Shape		Durbin-Watson	Maximum Cook's Distance
	Variable	Statistic	df	Significance	Skewness	Kurtosis	Statistic	Statistic
1	PTS/MIN	0.897	2,180	0.000	0.817	1.274	1.482	0.089
2	PTS/MIN	0.991	4,797	0.005	0.027	−0.161	1.801	0.373
3	PTS/MIN	0.990	1,725	0.017	0.133	0.017	1.785	0.121
4	PTS/MIN	0.994	3,642	0.002	0.027	0.089	1.621	0.124
5	PTS/MIN	0.987	888	0.001	0.213	0.430	1.843	0.120
6	PTS/MIN	0.978	2,883	0.000	0.819	1.688	1.810	0.721
7	PTS/MIN	0.989	2,004	0.000	0.569	1.269	1.759	1.032
8	PTS/MIN	0.982	2,074	0.000	0.399	0.478	1.604	0.448
9	PTS/MIN	0.996	2,308	0.000	0.110	0.160	1.674	0.244

Note: PTS/MIN, points per minute.

Unique regression models, refined by retaining only statistically significant variables, were developed for each cluster. These models were then used to predict player “points per minute”, enabling a relative ranking of players within their respective archetypes (see Table 7). To account for variations in competitive strength, a league quality weighting system was applied to these rankings (see Table 8). The ranking allows one to build the best roster composition, rather than just selecting the best available player, by understanding who the best players in each cluster were to be selected. Basketball is a game based on gaining more points than your opponent; therefore, one must still select the best players to get the points without ruining the roster composition.

**Table 7:** Regression lines for each archetype.

Cluster	Regression line
1	PTS/MIN = (−0.099640) + (MIN%)(0.000205) + (ORB/48)(−0.001765) + (PF/48)(−0.001489) + (FTA/48)(0.011766) + (FT%)(−0.000161) + (2PA/48)(0.015601) + (2P%)(0.001680) + (3PA/48)(0.017419) + (3P%)(0.001907)
2	PTS/MIN = (−0.277331) + (MIN%)(0.000090) + (AST/48)(0.000551) + (BLK/48)(0.002619) + (PF/48)(−0.000399) + (FTA/48)(0.016752) + (FT%)(0.000478) + (2PA/48)(0.018880) + (2P%)(0.001822) + (3PA/48)(0.021936) + (3P%)(0.004289)
3	PTS/MIN = (−0.268327) + (MIN%)(0.000135) + (DRB/48)(0.000695) + (STL/48)(0.001422) + (PF/48)(−0.00192) + (FTA/48)(0.013753) + (FT%)(0.000704) + (2PA/48)(0.020379) + (2P%)(0.002798) + (3PA/48)(0.017885) + (3P%)(0.002752)
4	PTS/MIN = (−0.249570) + (MIN%)(0.000242) + (DRB/48)(0.000752) + (AST/48)(0.000827) + (BLK/48)(0.003053) + (STL/48)(−0.001716) + (FTA/48)(0.014167) + (FT%)(0.000793) + (2PA/48)(0.021650) + (2P%)(0.003403) + (3PA/48)(0.020240)
5	PTS/MIN = (−0.329838) + (MIN%)(0.000106) + (ORB/48)(0.001345) + (DRB/48)(−0.000845) + (AST/48)(0.001506) + (BLK/48)(0.001975) + (STL/48)(−0.002208) + (FTA/48)(0.013718) + (FT%)(0.000752) + (2PA/48)(0.023044) + (2P%)(0.004757) + (3PA/48)(0.021283)
6	PTS/MIN = (−0.448741) + (MIN%)(0.000068) + (STL/48)(−0.001116) + (PF/48)(−0.000859) + (FTA/48)(0.016034) + (FT%)(0.001056) + (2PA/48)(0.020490) + (2P%)(0.004835) + (3PA/48)(0.021446) + (3P%)(0.003879)
7	PTS/MIN = (−0.461385) + (ORB/48)(−0.005009) + (DRB/48)(0.001619) + (AST/48)(−0.000984) + (STL/48)(−0.001829) + (FTA/48)(0.013037) + (FT%)(0.001435) + (2PA/48)(0.023181) + (2P%)(0.006911) + (3PA/48)(0.018835)
8	PTS/MIN = (−0.277067) + (MIN%)(0.000197) + (FTA/48)(0.012906) + (FT%)(0.000642) + (2PA/48)(0.021599) + (2P%)(0.004313) + (3PA/48)(0.013204)
9	PTS/MIN = (−0.213109) + (MIN%)(0.000216) + (ORB/48)(−0.002770) + (PF/48)(−0.002283) + (FTA/48)(0.014132) + (FT%)(0.000977) + (2PA/48)(0.018574) + (2P%)(0.003077) + (3PA/48)(0.022080)

**Table 8:** Competition ranking multiplier.

Competition	Weight
NBA Playoffs	2.00
G-League Playoffs	1.80
EuroLeague	1.90
EuroCup	1.80
Basketball Champions League	1.75
Europe Cup	1.60
Asia Champions Cup	1.60
Terrific 12	1.40
Summer 8	1.20
American League	1.55
South American League	1.50
NCAA Div I March Madness	1.70
NCAA Div II March Madness	1.35
U-Sports Elite 8	1.60

Note: North American Championships = NBA Playoffs, G-League Playoffs; European Championships = EuroLeague, EuroCup, Basketball Champions League, Europe Cup; Asian Championships = Asia Champions Cup, Terrific 12, Summer 8; Central and South American Championships = American League, South American League; University Championships = NCAA Div I March Madness, NCAA Div II March Madness, U-Sports Elite 8.

### 3.4 Optimal team archetype proportions

Optimal player cluster proportions for national team composition were derived from an analysis of 2018/19 NBA lineups and international medal-winning athletes from competitions between 2014 and 2018, selected based on pre-defined criteria. This analysis identified 320 relevant NBA players and 324 international athletes (see Table 9).

**Table 9:** Percentage of players’ clusters: Top NBA line-Ups and international medalists.

Cluster	Top NBA line-ups (*n* = 320)		International medallists (*n* = 324)		Total (*n* = 644)	
	Count	Percent in line-ups	Count	Percent in line-ups	Count	Percent in line-ups
1	3	0.9%	28	8.6%	31	4.8%
2	61	19.1%	61	18.8%	122	18.9%
3	1	0.3%	19	5.9%	20	3.1%
4	46	14.4%	43	13.3%	89	13.8%
5	13	4.1%	3	10.2%	46	7.1%
6	93	29.1%	57	17.6%	150	23.3%
7	56	17.5%	12	3.7%	68	10.6%
8	2	0.6%	44	13.6%	46	7.1%
9	45	14.1%	27	8.3%	72	11.2%

Based on the observed distributions within these highly successful team line-ups, the optimal team composition was determined. The analysis revealed that successful rosters consistently feature a balanced distribution of archetypes, emphasizing specific roles crucial for modern basketball success. Specifically, top-performing teams demonstrated a higher proportion of players from the “Aggressive Scorer”, “High Efficiency Scorer”, and “Floor General” archetypes, or clusters 6, 7, and 9, respectively. Conversely, archetypes such as “Low Efficiency Defender”, and “Fouler”, or clusters 1, and 8, respectively, were minimally represented or entirely absent.

The recommended optimal distribution of archetypes for a 15 player roster is as follows: 0–1 “Low Efficiency Defender”, 2–3 “Catch-and-Shoot Shooter”, 0–1 “3-and-D”, 2–3 “Athletic Shooter”, 1–2 “Aggressive Shot-Blocker”, 3–4 “Aggressive Scorer”, 2–3 “High Efficiency Scorer”, 0–1 “Fouler”, and 2–3 “Floor General”, adjusted by the coach as needed. This distribution prioritizes offensive efficiency, defensive versatility, and playmaking, which were identified as key drivers of team success in the data set. While specific numbers can vary slightly based on coaching philosophy and player availability, these proportions represent a general guideline for maximizing team effectiveness based on the identified player archetypes.

### 3.5 Canadian national team selection for the 2019 FIBA basketball world cup

The derived cluster proportions guided the selection of Canadian players, with a weighting towards NBA proportions in cases of close proximity between cluster allocations.

The constructed Canadian roster comprised 15 players selected based on the established proportionality within top teams and player clusters. The distribution across archetypes was: 0 players from cluster 1, 3, and 8; 2 players from cluster 4, 5, 7, and 9; 3 players from cluster 2; and 4 players from cluster 6. Notably, Canada possessed the second-largest pool of NBA athletes globally, after the United States (see Table 10). The model accurately predicted the selection of the majority of players for the national team, with the exception of Conor Morgan, Brandon Clarke, Mfiondu Kabengele, and Andrew Rautins, which was adjusted by Canadian coach preference to Khem Birch, Tristan Thompson, Cory Joseph, and Justin Jackson (see Table 11).

**Table 10:** Top Canadian players.

Cluster	Top 5 available Canadian players				
1	Alexandre Leclerc	Alex Thielen	Will Spaulding	Sterling Simpson	Dalano Banton
2	Dillon Brooks	Nik Stauskas	Andrew Rautins	Kassius Robertson	Kai Williams
3	Caleb Agada	Mathieu Kamba	Will Fiander	Tyrell Leotaud	Ibrahima Sylla
4	Kelly Olynyk	Conor Morgan	Dyshawn Pierre	MiKyle McIntosh	Aleksandar Danilovic
5	Chris Boucher	Brandon Clarke	Khem Birch	Drew Urquhart	Matt Neufeld
6	Jamal Murray	Andrew Wiggins	Trey Lyles	R.J. Barrett Jr.	Kyle Wiltjer
7	Dwight Powell	Mfiondu Kabengele	Dallin Bachynski	Tristan Thompson	Owen Klassen
8	Jordan Roinson	Warame Mohamed	Luka Zaharijevic	Jaes Karnik	Ryan Wright
9	Shai Gilgeous-Alexander	Kevin Pangos	Philip Scrubb	Trae Bell-Haynes	Xavier Rathan-Mayes

**Table 11:** Team Canada roster selection recommendation.

Data-based roster				Coach-based roster			
Team Position	Player	Cluster	PTS/MIN	Team Position	Player	Cluster	PTS/MIN
Starting five	Jamal Murray	6	0.552	Starting five	Jamal Murray	6	0.552
Starting five	Dillon Brooks	2	0.410	Starting five	Dillon Brooks	2	0.410
Starting five	Shai Gilgeous-Alexander	9	0.391	Starting five	Shai Gilgeous-Alexander	9	0.391
Starting five	Dwight Powell	7	0.503	Starting five	Dwight Powell	7	0.503
Starting five	Kelly Olynyk	4	0.470	Starting five	Kelly Olynyk	4	0.470
Bench	R.J. Barrett Jr.	6	0.672	Bench	R.J. Barrett Jr.	6	0.672
Bench	Brandon Clarke^a^	5	0.415	Bench	Khem Birch^a^	5	0.322
Bench	Andrew Wiggins	6	0.506	Bench	Andrew Wiggins	6	0.506
Bench	Trey Lyles	6	0.495	Bench	Trey Lyles	6	0.495
Bench	Conor Morgan^a^	4	0.439	Bench	Tristan Thompson^a^	7	0.405
Bench	Chris Boucher	5	0.812	Bench	Chris Boucher	5	0.812
Bench	Nik Stauskas	2	0.389	Bench	Cory Joseph^a^	9	0.282
Injury	Kevin Pangos	9	0.495	Injury	Kevin Pangos	9	0.495
Injury	Mfiondu Kabengele^a^	7	0.667	Injury	Justin Jackson^a^	2	0.259
Injury	Andrew Rautins^a^	2	0.480	Injury	Nik Stauskas	2	0.389

Note: ^a^Replacements made via coach's decision.

## 4 Discussion

### 4.1 Main findings and model utility

This study successfully identified nine distinct clusters of basketball players through comprehensive analysis of box score statistics across 110 leagues worldwide. While the inherent nature of cluster analysis means that definitive stability cannot be entirely guaranteed, the developed model offers a valuable framework for coaches and general managers to identify potential talent fits for their team, not only within their league but crossing over into other leagues. This tool is not intended as a definitive solution but rather as a starting point for strategic decision-making in player evaluation and team construction. For instance, NBA general managers could adapt this approach to inform player selection during NBA drafts from the NCAA and European leagues. Since player statistics would not necessarily be equal, they would be leveraging data-driven insights into player archetypes and potential. The study underscores the importance of integrating human judgment with analytical tools, aligning with the concept of collective intelligence to address data gaps (20). As Daryl Morey, a leader in basketball analytics, has stated, the true advantage of data lies in unique insights and the recognition that models are not a substitute for human expertise (21).

Many athletes analyzed in this model likely operate within a mastery-oriented environment, characterized by efforts to improve their skill sets (22). The motivational climate, defined as the psychological environment a coach creates to foster learning and motivate athletes (23), significantly influences player development (24). This model's emphasis on specific box score statistics correlates with training opportunities for enhanced skill development, supporting athlete progression to higher levels of competition. The intrinsic motivation for skill improvement (25–29) often drives mastery-oriented athletes forward, while more ego-focused athletes may be phased out. The model's highest-ranked players, predominantly from the NBA and high-level international leagues, exemplify the emphasis on skill mastery over ego in sustaining optimal motivation levels among professional athletes (30). For coaches, this ranking would help them quantify the potential of an athlete more effectively.

Professionals involved in talent evaluation, including scouts, coaches, and general managers, require robust methods to differentiate and quantify overall potential and actual quality of players across various levels of play. Previous models (31, 32) utilized athletic variables such as defensive help and screening efficiency, whereas the current model employed more objective box score statistics like “points per 48 min”. Given the significant role coaches play in athlete orientation and development (9), this model provides a method for classifying players based on specific attributes indicative of potential to advance to higher competitive levels. The ability to distinguish between a player's potential quality, based on physical characteristics, and actual quality, based on on-court results (9, 10), is crucial to avoid premature labeling. The model also aids in identifying “green bananas”, athletes who exhibit delayed development but possess significant long-term potential. By utilizing accessible and broad-based variables, the model supports enhanced scouting efforts to identify “diamond in the rough” players and maximize the potential for future growth and success by ensuring athletes possess diverse skill sets, particularly as the NBA expands its global reach.

The model's quality was further assessed by observing how the ranking system accounted for external factors. While factors like home court advantage, shot location, and shot types positively impact player performance (16, 33, 34), and positional differences in performance are evident (17), these were addressed through the developed league weighting system and player ranking. Similar ranking systems are employed by organizations such as the International Basketball Federation (11) and the Union of European Football Association (12) for national teams and leagues, respectively. By combining optimal descriptive player variables with a uniform league ranking system, the model effectively assesses a player's true value through the identification of distinct player archetypes, without being biased by the league of participation, which can lead to improved outcomes in player drafting and placement in optimal developmental situations.

Future refinements to the model, particularly for high-level competitions like the NBA or Olympics, could involve excluding players and teams from International Pro B and C leagues to further enhance clustering and regression accuracy, especially since none of the teams in those leagues competed in high-level championships. Discussions with Canada's national team coaches highlighted that certain non-quantifiable factors, such as senior leadership, are not always translatable to a statistical model. This difference represents a crucial area where human expertise complements data-driven insights. For example, coaching staff might select senior players like Cory Joseph, Khem Birch, and Tristan Thompson for leadership despite potentially lower points per minute statistics or opt for promising talents like Justin Jackson who may struggle in their rookie seasons but are valued for long-term potential. Notably, the model's deviation from selecting established veterans in favour of promising talents like Mfiondu Kabengele and Brandon Clarke, both 2019 NBA 1st round draft picks, demonstrates its ability to identify future high-potential players.

The model's utility extends to understanding player availability and team construction across different national contexts. For instance, the United States, with its substantial NBA talent pool of 453 Americans in the NBA in 2024/25 compared to 25 Canadians, may not need to seek players from other leagues. However, for specific competitions, top players might decline participation or require rest. At the 2023 FIBA World Cup, the United States roster did not include 8 of the top 10 American scorers, and included Chet Holmgren, who had not yet played in the NBA. This model can address player availability regardless of a singular focus on leagues like the NBA. Furthermore, the model can illustrate player adaptability. For the 2024 Olympics, the United States roster included 6 “aggressive scorer” archetypes, including Devin Booker. His subsequent adaptation to a “floor general” role, where higher 2-point percentage, 3-point percentage, and assists per 48 min were needed, highlights how the model not only identifies archetypes but can also inform coaching and scouting to help players adapt to team needs and future opportunities.

### 4.2 Application to Canadian national team performance: 2000, 2024, and beyond

To further evaluate the model's effectiveness, the 2000 Canadian Olympic team roster was analyzed against the model's predicted successful team proportions (see Table 12). The 2000 Canadian Olympic team, the last Canadian men's team to qualify for the Olympics prior to Paris 2024, finished 7th. An analysis of the team's player composition revealed an over-representation of players from clusters 2, 3, 5, and 8, alongside an under-representation from clusters 1, 4, 6, 7, and 9 (see Table 13). This observed discrepancy between the actual team composition and the model's predicted proportions suggests that the model effectively identified areas for improvement in the team's selection process, reinforcing its utility.

**Table 12:** Team Canada 2000 Olympic roster.

Team position	Player	Cluster	PTS/MIN
Starting five	Steve Nash	2	0.427
Starting Five	Sherman Hamilton	3	0.244
Starting five	Pete Guarasci	5	0.434
Starting five	Michael Meeks	6	0.588
Starting five	Todd Macculloch	5	0.595
Bench	David Daniels	4	0.190
Bench	Andrew Mavis	3	0.206
Bench	Rowan Barrett	2	0.461
Bench	Eric Hinrichsen	5	0.167
Bench	Greg Francis	2	0.467
Bench	Gregory Newton	8	0.452
Bench	Shawn Swords	8	0.149

**Table 13:** Team selection quality in the last Olympic games Canada participated.

Cluster	Team Canada 2019	Team Canada 2000		
	Percent in Line-Ups	Count	Expected	Accuracy
1	4.8%	0	1	Under
2	18.9%	3	2	Over
3	3.1%	2	0	Over
4	13.8%	1	2	Under
5	7.1%	3	1	Over
6	23.3%	1	3	Under
7	10.6%	0	1	Under
8	7.1%	2	1	Over
9	11.2%	0	1	Under

Team Canada finished 7th at the 2000 Olympic Games.

The current model effectively identified the potential rise and success of several players. R.J. Barrett, Brandon Clarke, and Mfiondu Kabengele, drafted 3rd, 21st, and 27th respectively in the 2019 NBA draft, have since demonstrated success with Barrett and Clarke being in the NBA, and Kabengele being an All-EuroCup First Team selection. Jamal Murray played a prominent role in the 2023 NBA Championship, and Shai Gilgeous-Alexander, a rookie in 2019, has become the 2025 NBA Most Valuable Player (MVP), NBA Finals MVP, and scoring leader, 1 of 4 players in NBA history. Additionally, 7 out of 12 of the model's predicted players were eventually selected for the Team Canada roster that qualified for the 2024 Paris Olympic Games. The five players not identified in the initial model were Luguentz Dort, Nickeil Alexander-Walker, Melvin Ejim, Andrew Nembhard, and Khem Birch. Dort and Alexander-Walker were in the 2019 draft class, Nembhard was still a collegiate player with the University of Florida Gators, while Birch and Ejim were established veterans likely included for their experience. The former 3 players' archetypes likely evolved with development. In the context of the 2019 FIBA World Cup, Canada finished 21st in the group stage, with only Conor Morgan from this analysis being part of the actual Team Canada roster that competed. These decisions were based on availability from players, particularly players training for the NBA season, influencing team structure. However, this model can support and mitigate the effects of lack of player availability due to unforeseen circumstances. Ultimately, this model demonstrates potential for long-term utility and can be adjusted with different variables to improve its predictive accuracy.

### 4.3 Limitations and recommendations

Several limitations to the modeling approach may have impacted the initial clustering and subsequent ranking. For clustering, the inclusion of lower-tier leagues potentially introduced heterogeneity, as player talent levels and career aspirations may differ significantly from those in higher-level competitions. Additionally, data collection from multiple sources could introduce inconsistencies. For ranking, the league weighting system, based on subjective discussions, may not fully capture the nuanced relative importance of each league, thereby potentially influencing player rankings. However, despite these limitations, the model demonstrated notable effectiveness.

To improve the model, future refinements could include narrowing the scope of included leagues to those most relevant for identifying NBA or national-level talent. Incorporating more nuanced variables, and diversifying offensive and defensive metrics, could provide a more comprehensive understanding of each player's performance and overall contribution. Ultimately, while serving as a valuable tool for player evaluation, it is crucial to recognize that the model should not be considered a definitive solution, but rather a robust analytical aid.

## 5 Conclusion

### 5.1 Summary

This study aimed to determine the feasibility of clustering and ranking basketball players globally to effectively build championship teams. A comprehensive dataset of 22,500 elite professional and university athletes from the NBA, G-League, and international leagues was compiled from EuroBasket, RealGM, and USportsHoops. Player performance was quantified using 13 key box score statistics, standardized to a per 48 min basis, for subsequent clustering.

Following data standardization, players were clustered into nine distinct archetypes. Each cluster was then analyzed using multiple linear regression to identify the key variables contributing to “points per minute” for that archetype, enabling player ranking. The optimal proportion of each cluster within successful NBA lineups and international medal-winning teams was determined. Based on these findings and the model's player rankings, a hypothetical Team Canada roster was selected for the 2019 FIBA World Cup. The effectiveness of the clustering and player ranking was evaluated by comparing the selected roster to the composition of past Canadian national teams and their subsequent performance, followed by a retrospective review of selected players five years post-modeling. The model demonstrated effectiveness in identifying a robust roster for Team Canada's 2019 FIBA World Cup participation.

### 5.2 Practical implications

This research yields several practical implications for players, coaches, scouts, and general managers. For players and coaches, a comprehensive league metric was developed, incorporating a unique variable derived from competition results, providing a robust measure of league strength that can inform future career pathways. For scouts and general managers, a novel player ranking system, established through the integration of cluster analysis and regression analysis, offers a sophisticated and nuanced evaluation of player performance for optimal team construction. The inclusion of an extensive dataset encompassing 22,500 players from around the world provides a unique opportunity to analyze and understand the global landscape of basketball talent.

## Data Availability

The datasets presented in this article are not readily available because hard drive containing the data was erased. Data is available but it is extensive and doesn't have the equations. Requests to access the datasets should be directed to lukepennerbasketball@gmail.com.
